# DAQ-YOLO:a high precision counting model for maize seedlings in dense scenes

**DOI:** 10.3389/fpls.2026.1879331

**Published:** 2026-09-03

**Authors:** Xiongwei He, Haonan Wang, Zhenyu Ma, Kangjie Li, Tiantian Zhou, Yongqiang Liu, Yingjie Zhou, Xiaoying Zhang

**Affiliations:** Faculty of Software Technologies, Shanxi Agricultural University, Jinzhong, China

**Keywords:** adaptive non-maximum suppression, dense scenes, dynamic attention, maize seedling counting, quality-aware focal loss

## Abstract

To address the problems of low detection accuracy, frequent missed detection and false detection in maize seedling counting tasks, this paper proposes a high-precision counting model for maize seedlings in dense scenes based on improved YOLOv8n, named DAQYOLO. Firstly, a dynamic and efficient multi-scale attention module is integrated into the neck network, which effectively alleviates the feature dilution of small targets in dense scenes and enhances the model’s ability to capture global and local information. Secondly, an adaptive quality-aware focal loss is adopted to perform collaborative optimization from the two aspects of classification accuracy and localization quality coupling, so as to improve the detection accuracy for overlapping and occluded targets in dense scenes. Finally, a quality-enhanced heterogeneous adaptive non-maximum suppression method is used to adaptively adjust the IoU threshold according to the density of target regions, which eliminates redundant detection boxes and improves counting accuracy simultaneously. Validated on a self-constructed dataset, experimental results indicate that—while maintaining a near-constant parameter—the proposed model outperforms the baseline YOLOv8n, with precision, recall, mAP50 and mAP50–95 increases by 2.0%, 3.3%, 2.1% and 8.0%, respectively. This method provides a technical foundation for efficient and accurate counting of maize seedlings.

## 1 Introduction

As one of the world’s important food crops, maize has maintained a steady growth trajectory in both cultivation area and total yield, holding profound strategic significance in ensuring global food security, livestock feed production and diverse industrial applications (Erenstein et al., 2022; Padhan et al., 2024; Shiferaw et al., 2011). With the rapid advancement of intelligent precision agriculture, the accurate quantification of maize seedlings has emerged as a fundamental basis for critical agricultural workflows, including high-throughput seed quality assessment (Wu et al., 2025), precise field management, dynamic seedling monitoring (Gao et al., 2023), and early-stage yield prediction (Danilevicz et al., 2021).

Conventionally, maize seedling counting has relied predominantly on manual inspection, which is inherently constrained by poor accuracy, low throughput, high labor costs, and significant subjectivity induced biases. Consequently, these manual approaches struggle to scale effectively for large-scale precision production and fall short of the rigorous real-time monitoring requirements mandated by modern smart agriculture (Jiang et al., 2026).

With the rapid development of deep learning technology, two-stage detectors, typified by Faster RCNN (Khan et al., 2025; Ren et al., 2017) and one-stage frameworks, such as SSD (Liu et al., 2016) and the YOLO series (Redmon et al., 2016; Kang et al., 2025; Wang et al., 2023, 2024a), have substantially bolstered detection precision and computational efficiency. Notably, the YOLO architecture has been extensively deployed in agricultural vision tasks due to its superior real-time inference and hardware friendly flexibility. As a contemporary mainstream framework, YOLOv8 (Ali and Zhang, 2024; Chen et al., 2024) offers a streamlined structure and robust generalization; its modular design facilitates customized optimizations tailored for maize seedling identification.

To augment feature representation, researchers have frequently incorporated attention mechanisms to enhance the model’s attention to subtle features. For instance, Woo et al. (2018) introduced the Convolutional Block Attention Module (CBAM), Yang et al. (2024) integrated a global attention mechanism into YOLOv8 to enhance the capture of micro-target details in complex farmland environments. Li et al. (2025a) employed Neighborhood Attention to strengthen phenotypic feature extraction, and the MA-YOLO model significantly alleviated the problem of insufficient feature representation for small agricultural targets by combining multi-scale feature fusion and attention mechanism (Lu et al., 2025). However, when processing morphologically similar and densely packed maize seedlings, conventional attention modules often incur prohibitive computational overhead, leading to degraded inference speeds and exhibit limited capacity for local detail preservation.

In terms of loss function optimization, to address class imbalance and the varying difficulty of sample learning, Zhang et al. (2022) adopted EIoU Loss to accelerate bounding box convergence, Wu et al. (2024) introduced WIoU to optimize anchor quality through a dynamic focusing mechanism. Li et al. (2025b) proposed the use of Unified Intersection-Union Ratio Loss to optimize bounding box localization, Wise-IoU (Liu et al., 2025) and CIoU (Liu et al., 2023), have been explored to facilitate convergence and effectively reduce the aspect ratio penalty. To address the occlusion problem in crowded scenes, Wang et al. (2018) proposed a novel bounding-box regression loss specifically designed for crowd detection, termed Repulsion Loss. Nevertheless, in high-density planting scenarios characterized by severe occlusion, standard loss refinements struggle to simultaneously harmonize precise localization and classification confidence for overlapping targets.

Regarding post-processing, traditional Non-Maximum Suppression (NMS) is prone to erroneously suppressing true positive candidates in crowded scenes (Noh et al., 2024; Liu et al., 2019). Although Soft-NMS (Bodla et al., 2017) and DIoU-NMS (Zheng et al., 2020) attempt to retain overlapping boxes or reduction of redundant frames based on center point distance, they often lack adaptive thresholding. Solovyev et al. (2021) proposed a weighted box fusion algorithm is, The algorithm leverages the confidence scores of all candidate bounding boxes to perform a weighted average of bounding box coordinates, thereby generating a new fused box. For dense crowd detection. Wu and Yang (2023) proposed a region-adaptive non-maximum suppression (NMS) threshold and a decouple-then-align paradigm to address the limitations of existing pedestrian detection methods. However, in extremely high-density maize seedling distributions, these strategies remain susceptible to missed detection and false detection.

Although current research has achieved favorable results in object detection, the task of counting maize seedlings is hindered by factors such as small target size, similar morphological characteristics, dense distribution, and occlusion, which significantly increase the difficulty of distinguishing individual targets. To address the limitations of existing studies, this paper takes the counting of maize seedlings in greenhouses as the research object, uses a self-built maize seedling dataset, and proposes a high precision detection model named DAQ-YOLO based on YOLOv8n. By introducing a dynamic and efficient multi-scale attention module, an adaptive quality-aware focal loss function, and a quality-enhanced heterogeneous adaptive non-maximum suppression method, the model significantly improves the counting accuracy of maize seedlings.

The main contributions of this study are summarized as follows:

1. A novel counting model for maize seedlings DAQ-YOLO is proposed. This model integrates a dynamic and efficient multi-scale attention module into its neck network. Through dynamic channel grouping and weighted residual structures, the module effectively alleviates the feature dilution issue of small targets in dense scenes while maintaining the model’s lightweight nature, and enhancing the model’s ability to capture both global and local information.
2. In terms of loss function optimization, an adaptive quality-aware focal loss is adopted to perform collaborative optimization from two aspects: classification accuracy and localization quality coupling. By using a dynamic modulation factor to suppress gradient updates dominated by easily identifiable samples and through introducing a localization quality weight factor, different weights are assigned to easy and hard samples, it reduces the localization deviation for hard-to-identify samples, thereby improving the detection accuracy for overlapping and occluded targets in dense scenes.
3. In the post-processing stage, a quality-enhanced heterogeneous adaptive non-maximum suppression method is adopted. This method performs collaborative optimization through density-aware adaptive threshold learning and multi-dimensional quality-aware ranking suppression. It adaptively adjusts the IoU threshold according to the density of target regions, eliminating redundant detection boxes while improving counting accuracy.

## 2 Materials and methods

### 2.1 Dataset construction

**Figure 1:**
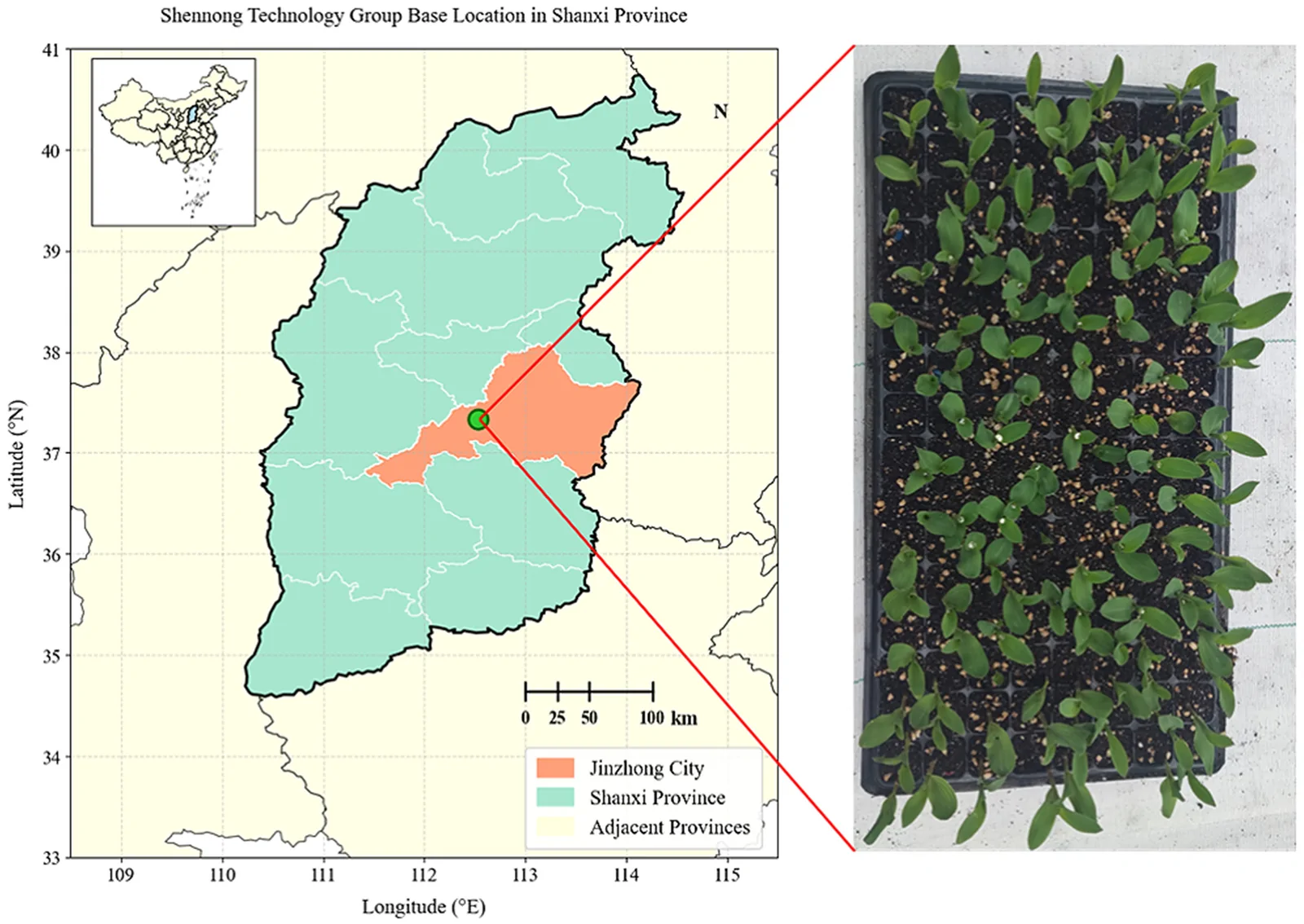
Map of actual sampling site coordinates.

The maize seedling dataset was collected at the greenhouse facility of the Northern China Forestry and Fruit Seedling Breeding Base, Shennong Technology Group, located in Taigu District, Jinzhong City, Shanxi Province, China (37.337528°N, 112.533153°E). Data collection was conducted in two stages. In the first stage, the maize cultivar ‘Linong 368’ was used, and images were collected from April 18 to April 20, 2025, between 10:00 and 16:00 each day. In the second stage, the maize cultivars ‘Tieming 366’ and ‘Lanku 8165’ were used, and images were collected from June 21 to June 24, 2025, between 08:00 and 18:00 each day. All images were acquired using an iPhone 14 at a resolution of 4000×2250 pixels and saved in JPG format. The resulting dataset comprised 1,100 valid images of three maize cultivars captured under varying illumination conditions. The geographic distribution of the sampling site and representative image samples are illustrated in **Figure 1**.

### 2.2 Data preprocessing

**Figure 2:**
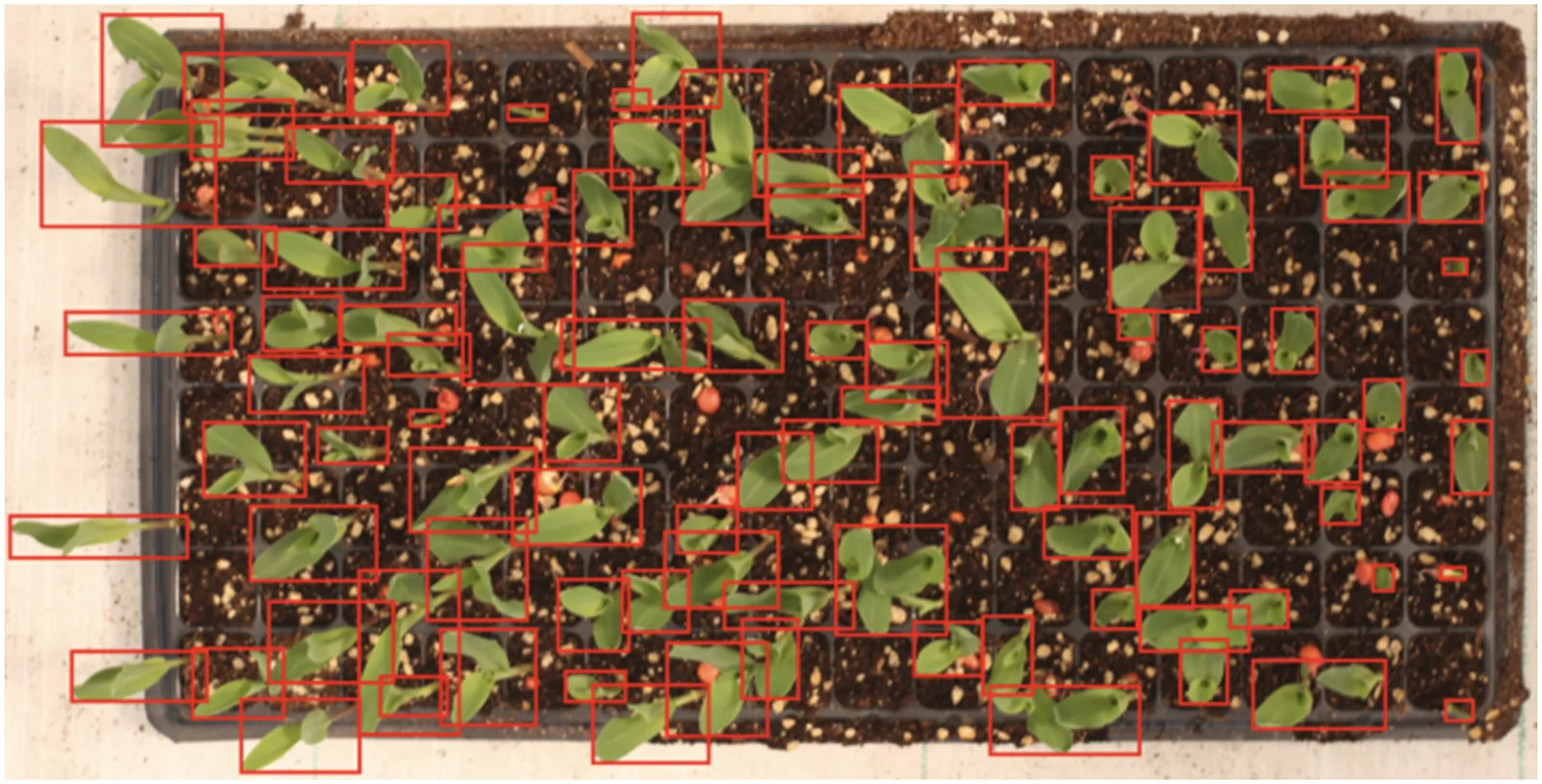
Annotated images of maize seedlings.

The experimental data are divided into training set, validation set and test set at a ratio of 8:1:1 according to different varieties, collection date and different light conditions. The actual partitioning results are shown in **Table 1**; different datasets contain images with distinct sequence numbers, thereby ensuring the independence of the three sets.To generate the ground truth for model supervision, all maize seedlings within the images were manually annotated using the LabelImg tool. The spatial coordinates and category information of each seedling were exported and archived in TXT format in accordance with the YOLO labeling convention. In total, 122651 individual maize seedling instances were meticulously labeled, ensuring a robust and high-density database for network training and performance evaluation. Sample annotated images of maize seedlings are shown in **Figure 2**.

**Table 1:** Details information of the maize seedling dataset.

DataSet	Variety	ID	Number	Label count
Training set	Linong 368	1-293	880	33809
	Tieming 366	294-586		31792
	Lanku 8165	587-880		32276
Validation set	Linong 368	881-916	110	4098
	Tieming 366	917-953		4252
	Lanku 8165	954-990		4086
Test set	Linong 368	991-1026	110	3978
	Tieming 366	1027-1063		4144
	Lanku 8165	1064-1100		4216
Total number	3	1-1100	1100	122651

### 2.3 Proposed DAQ-YOLO network

**Figure 3:**
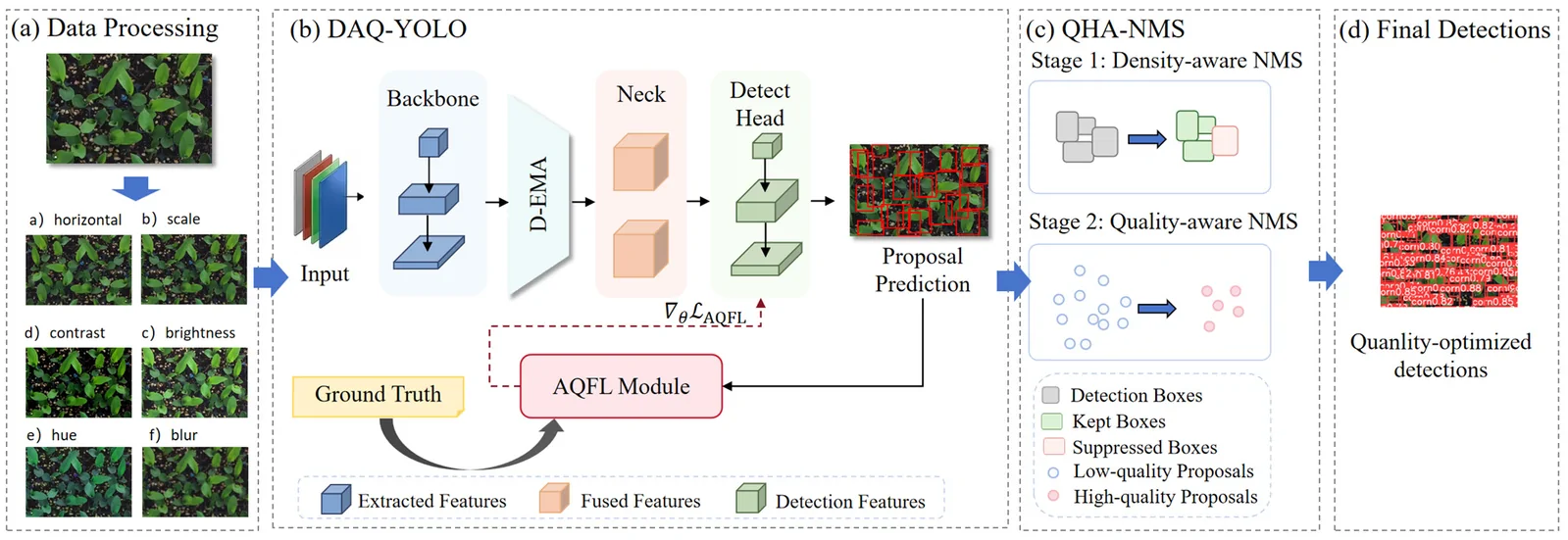
Logical structure of the proposed model.

**Figure 4:**
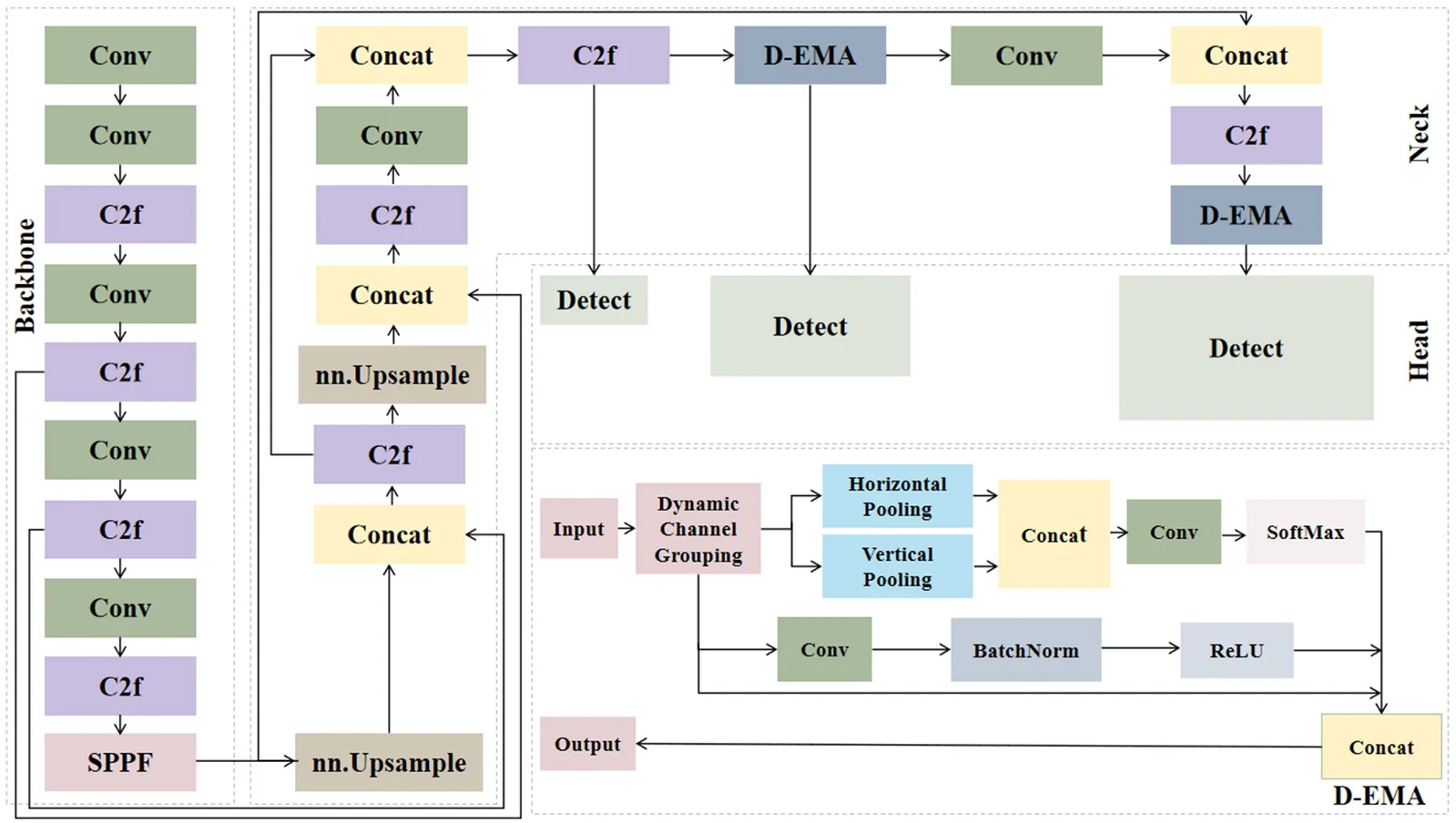
Network architecture of the DAQ-YOLO model.

In this study, an end-to-end detection framework centered on multi-module collaborative optimization is proposed, building on the YOLOv8n baseline architecture (Terven et al., 2023). The framework integrates three core components: Dynamic Efficient Multi-scale Attention (D-EMA) module, Adaptive Quality aware Focal Loss (AQFL) module, and Quality-Augmented Heterogeneous Adaptive Non-Maximum Suppression (QHA-NMS) module. These modules operate synergistically throughout the detection pipeline to optimize the process from feature extraction to post-processing. The logical structure and overall network architecture of the proposed model are elucidated in **Figures 3**, **4**, respectively.

#### 2.3.1 Dynamic efficient multi-scale attention module

In maize seedling counting tasks, small-scale targets are often characterized by significant scale fluctuations, weak local textures, and uneven spatial distribution. Relying solely on the convolutional stacking of the backbone network makes it difficult to simultaneously balance spatial sensitivity and channel representation efficiency (Wang et al., 2025). While a single attention mechanism is prone to redundant computation under high-channel features, fixed grouping strategies often demonstrate instability across hierarchical feature maps (Wang et al., 2019). To mitigate these limitations, we designed the

**Figure 5:**
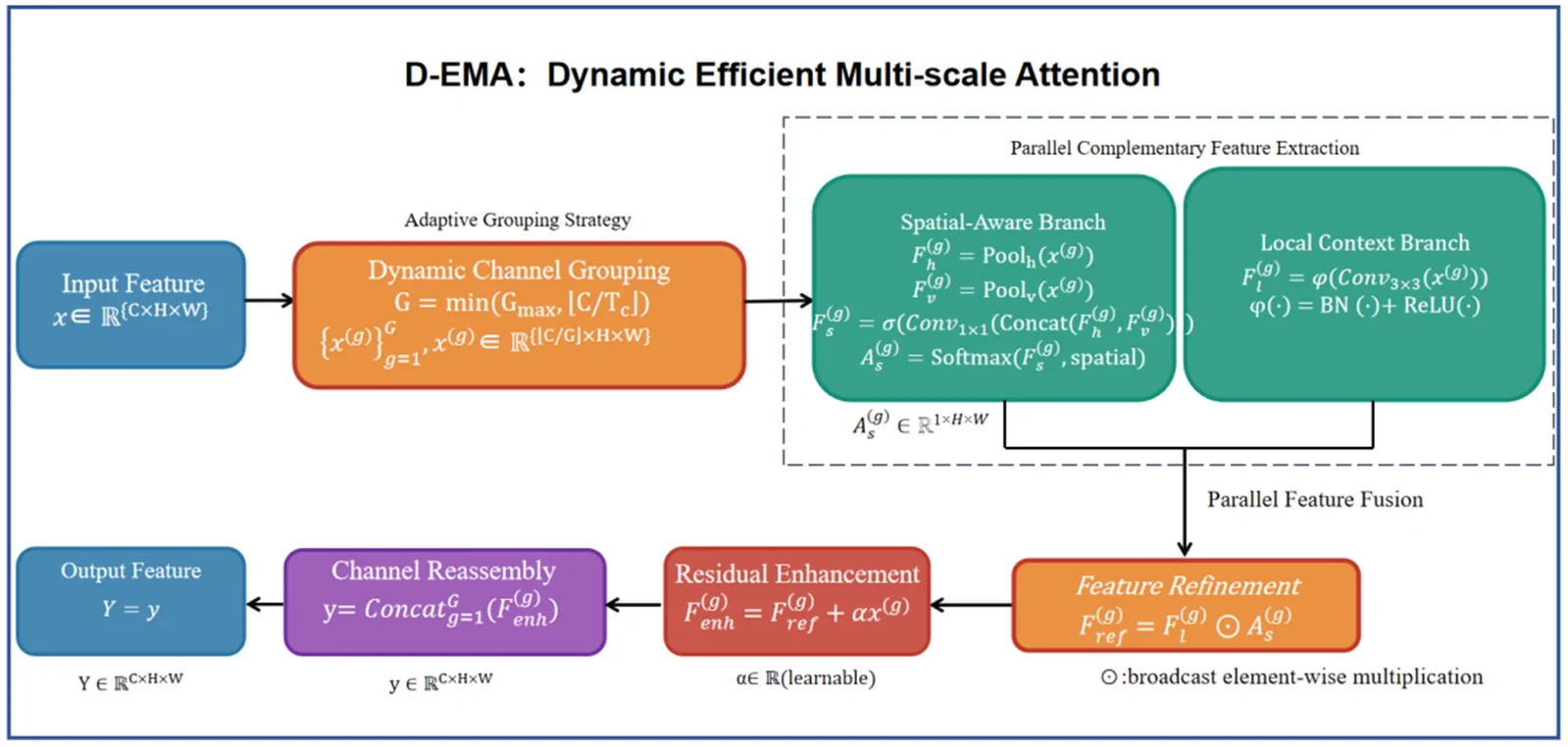
Structure of the D-EMA module.

Dynamic Efficient Multi-scale Attention (D-EMA) module. Its core objective is not the simple stacking of attention structures, but rather the introduction of an adaptive spatial-channel collaborative modeling mechanism while strictly controlling computational complexity. The overall structure is illustrated in **Figure 5**, and the module can be divided into four functional components. The mathematical formulation of the D-EMA module is presented in **Equations 1**–**11**. 1. Dynamic Channel Grouping Given the input features:

(1) $$X\in {\mathbb{R}}^{C\times H\times W}$$

If spatial attention is applied directly across the full channel dimension, the computational overhead increases rapidly as the channel count grows (Hu et al., 2018); conversely, fixed channel grouping introduces over-segmentation issues in low-channel layers (Wang et al., 2020). To mitigate this, the number of groups G is no longer manually pre-defined but is dynamically determined based on the channel scale (Su et al., 2020).

(2) $$G=\text{min }\left(G_{\text{max }\;},\;\lfloor C/T_{c}\rfloor \right)$$

Where *G*_max_ controls the maximum grouping scale, and *T_c_* is utilized to limit the lower bound of channels within a single group. This strategy enables the number of groups to adjust dynamically in response to variations in feature dimensions, thereby maintaining a stable intra-group representational capacity across different hierarchical levels. The features are then partitioned as:

(3) $${\left\{x^{\left(g\right)}\right\}}_{g=1}^{G},\;\;x^{\left(g\right)}\in {\mathbb{R}}^{C/G\times H\times W}$$

The primary objective of this decomposition is not dimensionality reduction but rather to provide a more compact subspace representation for subsequent local spatial modeling.

2. Parallel Complementary Feature Extraction

When relying solely on local convolutions to enhance spatial distribution, attention responses tend to become overly smooth; conversely, pure spatial pooling inevitably weakens local structural information (Gao et al., 2019). To address this, parallel branching is employed instead of cascading. Within each subspace, two complementary paths are constructed in parallel.

Spatial-Aware Branch: Global pooling is executed along the horizontal and vertical directions, respectively (Hou et al., 2021).

(4) $$F_{h}^{\left(g\right)}=\underset{\left(h\right)}{\text{Pool}}\;\left(x^{\left(g\right)}\right)$$

(5) $$F_{v}^{\left(g\right)}=\underset{\left(v\right)}{\text{Pool}}\;\left(x^{\left(g\right)}\right)$$

Horizontal pooling captures vertical variations, while vertical pooling captures horizontal variations. Subsequently, a 1×1 convolution is applied for feature fusion:

(6) $$F_{s}^{\left(g\right)}=\sigma \;\left(\underset{1\times 1}{\text{Conv}}\;\left(\text{Concat }\left[F_{h}^{\left(g\right)},\;F_{v}^{\left(g\right)}\right]\right)\right)$$

Where *σ* denotes the Sigmoid activation function, and Concat represents the concatenation operation. Finally, a normalization operation is performed to obtain the spatially enhanced feature feature $A_{s}^{\left(g\right)}$:

(7) $$A_{s}^{\left(g\right)}=\text{Softmax }\left(F_{s}^{\left(g\right)},\text{ spatial}\right)$$

Where $A_{s}^{\left(g\right)}\in {\mathbb{R}}^{1\times H\times W}$. This decomposed modeling approach enables the spatial weights to be aggregated separately along the row and column directions prior to fusion, which is more conducive to preserving directional information than direct 2D pooling.

Local Context Branch: Standard convolutions are employed within the subspace to extract local contextual information in parallel.

(8) $$F_{l}^{\left(g\right)}=\phi \;\left({\text{Conv}}_{3\times 3}\left(x^{\left(g\right)}\right)\right)$$

Where *ϕ*(·) = ReLU(BN(·)) denotes the composite operation comprising Batch Normalization (BN) followed by a non-linear ReLU activation. This sub-path focuses on capturing the local structural information of small-scale targets, thereby forming a complement to the spatial-aware branch. It utilizes a 3×3 convolutional kernel to extract local features, which preserves spatial structural information while simultaneously enhancing channel representational capacity.

3. Feature Refinement

The spatial attention weights are utilized to modulate the local contextual features:

(9) $$F_{\text{ref}}^{\left(g\right)}=F_{l}^{\left(g\right)}⊙A_{s}^{\left(g\right)}$$

Where ⊙ denotes broadcast element-wise multiplication. In contrast to conventional channel attention mechanisms, the attention here is applied exclusively across the spatial dimensions, thereby avoiding the information loss associated with additional channel compression.

4. Residual Enhancement and Channel Reassembly

Relying solely on attention enhancement may attenuate the original response intensity in certain scenarios. To mitigate this, a weighted residual structure is introduced (He et al., 2016), dynamically modulating the branch contributions via learnable parameters (Ding et al., 2021).

(10) $$F_{\text{enh}}^{\left(g\right)}=F_{\text{ref}}^{\left(g\right)}+\alpha \;x^{\left(g\right)}$$

Where *α* denotes a learnable parameter utilized to regulate the proportional relationship between the original features and the enhancement branch.

Finally, the respective feature groups are concatenated along the channel dimension, as formulated below, yielding the output $y\in {\mathbb{R}}^{C\times H\times W}$:

(11) $$y={\text{Concat}}_{g=1}^{G}\;\left(F_{\text{enh}}^{\left(g\right)}\right)$$

Compared with the traditional EMA, the design of D-EMA transcends the mere addition of attention branches by introducing enhancements across three dimensions: first, the number of groups is dynamically determined to prevent the insufficient adaptability of fixed grouping strategies to multi-scale features; second, spatial and local contexts are modeled in parallel rather than through cascaded enhancement; third, the residual weights are learnable, preventing the attention mechanism from excessively suppressing the original feature representations. Ultimately, the overall architecture maintains a lightweight profile while achieving an optimal balance between spatial sensitivity and channel utilization efficiency.

#### 2.3.2 Adaptive quality-aware focal loss module

The mathematical formulation of the AQFL module is presented in **Equations 12**–**27**. In deep learning, the network output is passed through the Sigmoid function to obtain the predicted probability p. Assume the label *m* ∈ {0,1}, then the model’s prediction confidence *p_t_*for the true category can be expressed as:

(12) $$p_{t}=\{\begin{array}{ll} p, & m=1\\ 1-p, & m=0 \end{array}$$

The traditional Focal Loss function can be formulated as:

(13) $$\text{FL }\left(p_{t}\right)=-\alpha_{t}{\left(1-p_{t}\right)}^{\gamma }\text{log }\left(p_{t}\right)$$

Where *γ* ≥ 0 is the focusing factor that controls the model’s attention on easy and hard samples; *α_t_*denotes the class balance factor used to mitigate the imbalance between positive and negative samples. The calculation formula of *α_t_*is defined as follows:

(14) $$\alpha_{t}=\{\begin{array}{ll} \alpha , & m=1\\ 1-\alpha , & m=0 \end{array}$$

In the experiments of the original paper, the hyperparameters are set as *γ* = 2, *α* = 0.25 (Lin et al., 2020).

**Figure 6:**
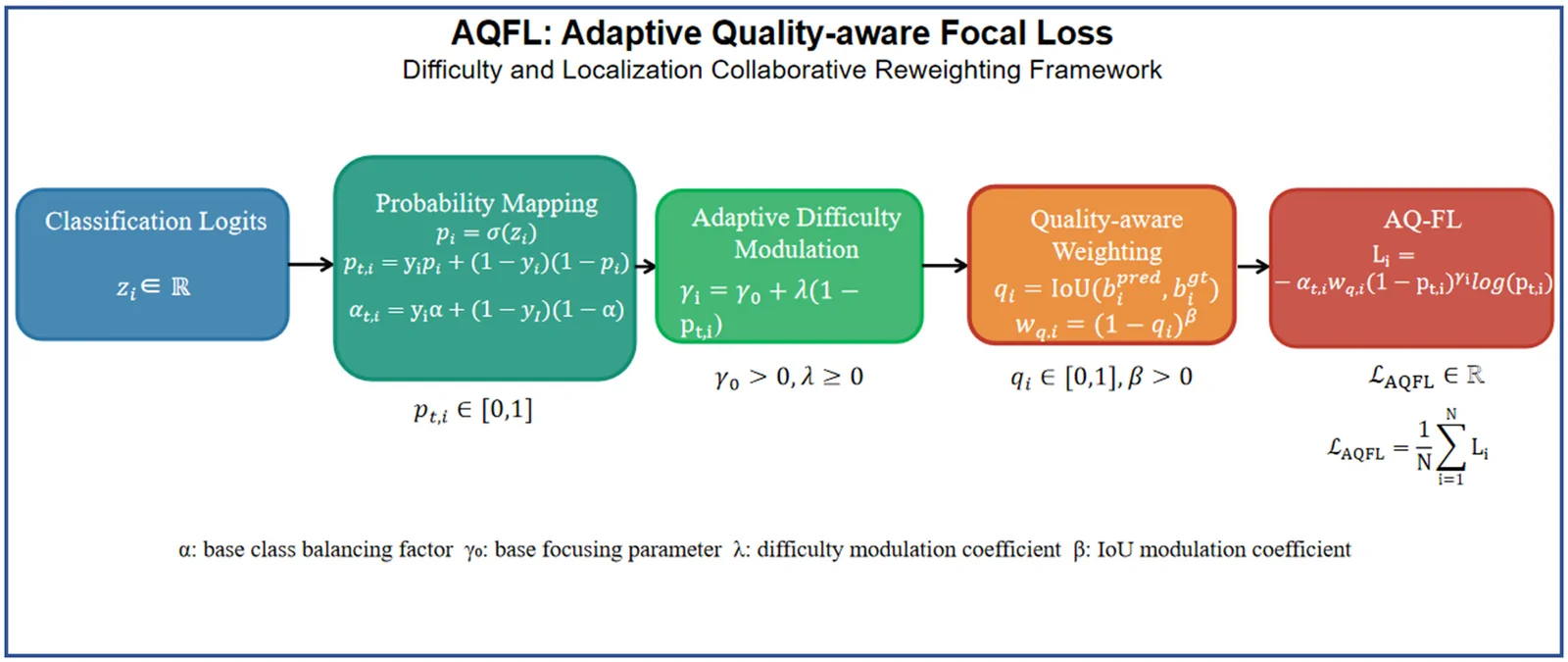
Structure of the AQFL module.

In dense small-target detection scenarios, the sample distribution exhibits pronounced imbalance characteristics. As a vast majority of easy-to-classify samples dominate the gradient updates (Wang et al., 2024c), challenging targets frequently suffer from significant localization deviations. While the traditional Focal Loss achieved the class balance through a fixed *α* and suppresses easily identifiable samples via a fixed focusing parameter *γ*, its suppression intensity remains constant throughout the training process, hindering adaptive adjustments based on actual sample difficulty. To address these limitations, an Adaptive Quality-aware Focal Loss (AQFL) is proposed in this paper. This approach achieves collaborative optimization by coupling classification accuracy with localization quality. The overall architecture of the AQFL module is illustrated in **Figure 6**.

For the maize seedling detection task studied in this work, maize seedling is the only positive category. During training, the network is encouraged to produce outputs approaching 0 or 1. This characteristic easily leads to overfitting and may cause misjudgments for hard samples. To address this issue, this paper introduces the concept of continuous soft labels. Instead of discrete values of 0 and 1, the labels adopt continuous values within the range [0,1]. By constraining the predicted outputs, soft labels alleviate overfitting and improve the model’s robustness for detecting maize seedlings with occlusion and blurred boundaries. The specific calculation procedure of continuous soft labels is described as follows.

1. Soft Label Generation Based on Task-Aligned Assigner

Task-Aligned Assigner:

This paper adopts the Task-Aligned Assigner proposed by Feng et al. (2021) to generate continuous soft labels. For the g-th ground-truth object and the i-th candidate anchor box, the task alignment metric is defined as:

(15) $$A_{g,i}=s_{i,c_{g}}^{\eta }\;u_{g,i}^{\tau }$$

Where g denotes the index of ground-truth objects, i denotes the index of candidate boxes, *c_g_* represents the category of the g-th ground-truth object; $s_{i,c_{g}}$ is the predicted probability of candidate box i for category *c_g_*; *u_g,i_* stands for the localization quality between the predicted box and the ground-truth box; *η* denotes the exponent of classification confidence, and *τ* denotes the exponent of localization quality. *u_g,i_* is defined by non-negative truncated CIoU:

(16) $$u_{g,i}=\text{max }\left[\text{CIoU }\left(b_{i}^{\text{pred}},\;b_{g}^{\text{gt}}\right),\;0\right],\;i\in \left\{1,2,\ldots ,K\right\}$$

Where $b_{i}^{\text{pred}}$ denotes the i-th predicted box; $b_{g}^{\text{gt}}$ denotes the g-th ground-truth box, and K represents the number of boxes selected with the highest overlap with the ground-truth object. In this work, the values of the classification confidence exponent *η*, localization quality exponent *τ*, and K follow the original paper, which are set to 0.5, 6.0 and 10, respectively (Jocher et al., 2023).

Let *P_g_* represent the set of positive samples assigned to the g-th ground-truth object. To alleviate the scale difference of the task alignment metric across various ground-truth targets, we normalize the task alignment metric as follows:


$${\tilde{A}}_{g,i}=\frac{A_{g,i}}{\underset{j\in P_{g}}{\max}\;A_{g,j}+\epsilon_{a}}\cdot \underset{j\in P_{g}}{\max}\;u_{g,j}$$

Where j is the index of candidate boxes belonging to set *P_g_*; ${\tilde{A}}_{g,i}$ denotes the normalized task alignment metric, and *ϵ_a_*= 10^−9^ is the numerical stability constant utilizede in the Task-Aligned Assigner to avoid division by zero.

Continuous Soft Label Generation:

Let *m* ∈ {0,1}, and g^*^(*i*) represent the ground-truth target matched ultimately with candidate box i. The classification soft label is defined as follows:

(18) $$y_{i,c}=\{\begin{array}{ll} {\tilde{A}}_{g^{*}\left(i\right),i}, & m=1\\ 0, & m=0 \end{array}$$

Where *y_i,c_* denotes the continuous soft label for category c, and *y_i,c_*∈ [0,1]. Since this work focuses on a single-class detection task with c=1, the soft label of positive samples equals the normalized task alignment metric. Accordingly, the formula can be simplified as $y_{i}={\tilde{A}}_{g^{*}\left(i\right),\;i}$ when *m* = 1, and *y_i_*= 0 when *m* = 0.

2. Prediction Probability and Class Balance

To uniformly represent positive samples, negative samples and continuous soft-label samples, the prediction probability can be expressed as Equation 19 (Li et al., 2020).

(19) $$p_{t,i}=y_{i}\;p_{i}+\left(1-y_{i}\right)\left(1-p_{i}\right)$$

This formula interpolates the positive-class probability and negative-class probability according to continuous soft labels, so as to represent positive samples, negative samples and soft-label samples in a unified form. When *y_i_*= 1, *p_t,i_* = *p_i_*; when *y_i_*= 0, *p_t,i_*= 1 − *p_i_*; when *y_i_*∈ (0,1), *p_t,i_* in this work is the interpolation result of the positive-class probability and negative-class probability. To avoid numerical overflow in logarithmic calculations when *p_t,i_*approaches 0, the practical implementation adopts:

(20) $$p_{t,i}\leftarrow \text{clip }\left(p_{t,i},\;\epsilon_{l},\;1-\epsilon_{l}\right),\;\epsilon_{l}=10^{-7}$$

In this work, the class balance weight is calculated using continuous soft labels, as shown in **Equation 21**. Compared with the fixed balance weights adopted in the original YOLOv8, the proposed method guarantees the continuity and smoothness of class balance weights for low-confidence samples, and improves training stability.

(21) $$\alpha_{t,i}=y_{i}\;\alpha_{f}+\left(1-y_{i}\right)\left(1-\alpha_{f}\right)$$

Where *α_f_* ∈ [0,1] denotes the base class balance coefficient of AQFL, and *α_f_* = 0.25 is set by referring to the original model. When *y_i_*= 1, *α_t,i_*= *α_f_*; when *y_i_*= 0, *α_t,i_*= 1 − *α_f_*. When *y_i_*∈ (0,1), the class balance weight varies continuously between the two values. This design keeps the weight consistent with the supervision strength of soft labels. It enables smooth interpolation of weights between the positive sample weight *α_f_* and the negative sample weight 1−*α_f_* according to continuous labels, instead of adopting discrete weights under binary label conditions.

3. Adaptive Focusing Parameter Modulation

The traditional Focal Loss adopts a fixed focusing parameter (gamma) RedTextto adjust the weights of hard samples. However, during practical training, sample difficulty dynamically changes with the model state, which makes the fixed (gamma) RedTextdifficult to effectively balance the optimization requirements at different training stages. To address this issue, this work constructs a sample-wise dynamic modulation factor.

(22) $$\gamma_{i}=\gamma_{0}+\lambda_{d}\left(1-p_{t,i}\right)$$

Where *γ*_0_ *>* 0 denotes the base focusing parameter, and *λ_d_*≥ 0 represents the modulation coefficient determined according to the difficulty of sample recognition. To constrain the modulation range, we set *λ_d_*= 1.0 in this work, and *γ*_0_ is set to 2.0 following the original model. When the sample confidence is low, *p_t,i_*becomes small, and the focusing factor increases automatically to strengthen the attention on hard samples. For samples with high confidence, the modulation strength declines, which helps maintain gradient stability. This mechanism overcomes the limitations introduced by a fixed *γ* and endows the training process with stronger adaptive capability.

4. IoU-Based Localization Quality Weighting

In dense target scenarios, relying solely on classification confidence is insufficient to accurately gauge prediction quality (Li et al., 2021). To strengthen the coupling relationship between classification and localization, an IoU-based quality weight is introduced (Zhang et al., 2021). In this work, the IoU between predicted boxes and ground-truth boxes is calculated for localization quality modulation. The localization quality is defined as:

(23) $$q_{i}=\{\begin{array}{ll} \text{IoU}\left(b_{i}^{\text{pred}},\;b_{i}^{\text{gt}}\right), & m=1\\ 0, & m=0 \end{array}$$

Where $b_{i}^{\text{pred}}$ denotes the i-th predicted bounding box, and $b_{i}^{\text{gt}}$ represents the matched ground-truth box corresponding to this positive sample. The localization quality weight is defined as:

(24) $$w_{q,i}={\left(1-q_{i}\right)}^{\beta_{q}}$$

Where *β_q_* ≥ 0 denotes the IoU quality modulation exponent. In this work *β_q_*= 1.0 is set, and the weight satisfies *w_q,i_*∈ [0,1]. When the overlap between the predicted box and ground-truth box is low, the corresponding weight increases, so that *w_q,i_*imposes stronger constraints on samples with insufficient localization quality. When the two boxes have a high overlap, the weight decreases naturally, which facilitates stable convergence. For negative samples, *q_i_*= 0 leading to *w_q,i_*= 1. Therefore, the IoUbased localization quality weighting introduces no additional scaling for negative samples, which are only regulated by the class balance term and focusing parameter modulation term.

5. Definition and Loss Normalization of AQFL

Combining class balance, adaptive focusing parameter modulation and localization quality weighting, the AQFL for the i-th candidate position is defined as:

(25) $$L_{i}=-\alpha_{t,i}\;w_{q,i}{\left(1-p_{t,i}\right)}^{\gamma_{i}}\log\left(p_{t,i}\right)$$

Where *α_t,i_*denotes the class balance weight; *w_q,i_*represents the localization quality weight; ${\left(1-p_{t,i}\right)}^{\gamma_{i}}$ is the dynamic Focal Loss modulation term, and -log(*p_t,i_*) stands for the basic classification loss. When the condition *λ_d_*= 0*, β_q_*= 0*, y_i_*∈ {0,1} is satisfied, then we have *γ_i_*= *γ*_0_ and *w_q,i_*= 1, and the loss function *L_i_*degenerates into the standard Focal Loss. The overall loss of AQFL is expressed as:

(26) $${ℒ}_{\text{AQFL}}=\frac{1}{N}\sum_{i=1}^{N}L_{i}=-\frac{1}{N}\sum_{i=1}^{N}\alpha_{t,i}\;w_{q,i}{\left(1-p_{t,i}\right)}^{\gamma_{i}}\log\left(p_{t,i}\right)$$

In the loss calculation of the proposed model, ${ℒ}_{\text{AQFL}}$ serves as the classification loss term. Together with the bounding box regression loss ${ℒ}_{\text{box}}$ and Distributional Focal Loss ${ℒ}_{\text{DFL}}$ adopted in the original model, it constitutes the total loss:

(27) $${ℒ}_{\text{det}}=\lambda_{\text{box}}{ℒ}_{\text{box}}+\lambda_{\text{cls}}{ℒ}_{\text{AQFL}}+\lambda_{\text{dfl}}{ℒ}_{\text{DFL}}$$

Where λ_box_, λ_cls_ and λ_dfl_ denote the gain coefficients for bounding box loss, classification loss and Distributional Focal Loss respectively. The values of these three parameters are consistent with those of the original model, which are 7.5, 0.5 and 1.5, respectively.

#### 2.3.3 Quality-augmented heterogeneous adaptive NMS module

**Figure 7:**
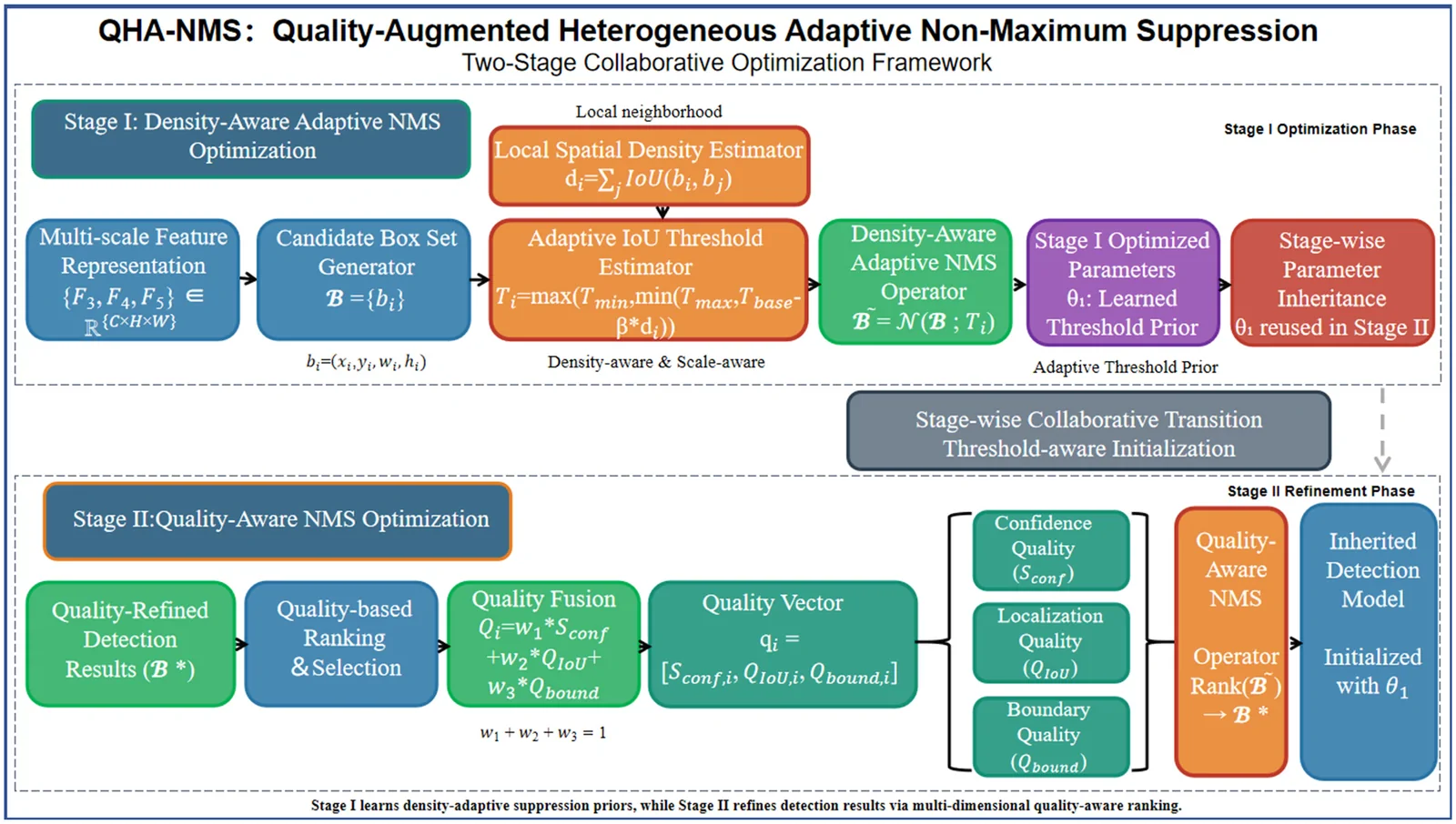
Overall structure of the QHA-NMS method.

In dense maize seedling scenarios, multiple candidate boxes with close positions and heavy overlaps tend to be generated around the same target. Conventional non-maximum suppression (NMS) generally sorts candidate boxes by classification confidence and eliminates redundant boxes using a fixed intersection-overunion (IoU) threshold. Nevertheless, a fixed threshold fails to adapt to variations in the overlap degree of candidate boxes across different regions, and classification confidence cannot fully reflect the spatial consistency and morphological rationality of candidate boxes. To address this issue, this paper proposes a Quality-Augmented Heterogeneous Adaptive NMS (QHA-NMS). The proposed method consists of two sequential stages. In the first stage, box-wise thresholds are calculated according to the overlap density of candidate boxes to implement preliminary filtering. In the second stage, multi-dimensional quality scores are adopted to re-determine the output priority of the boxes retained in the first stage. The overall structure is illustrated in **Figure 7**.

Let the candidate box set of QHA-NMS is *B*, $B={\left\{\left(b_{i},\;S_{\text{conf},i},\;c_{i}\right)\right\}}_{i=1}^{N}$, where N denotes the total number of candidate boxes; *b_i_*represents the i-th candidate box; *S*_conf_*_,i_* is the classification confidence with *S*_conf_*_,i_*∈ [0,1], and *c_i_*stands for the predicted category. QHA-NMS adopts a category-aware suppression strategy, meaning that mutual suppression is only performed between candidate boxes sharing the same predicted category. The mathematical formulation of the QHA-NMS module is presented in **Equations 28**–**34**.Stage I:

1. Density-Aware Adaptive Threshold Calculation

To characterize the distribution properties of local candidate bounding boxes, a local spatial density estimation function is defined as follows:

(28) $$d_{i}=\sum_{j}\text{IoU}\left(b_{i},\;b_{j}\right)$$

Where *d_i_* ≥ 0 denotes the overlap density score of candidate box *b_i_*; j represents the index of candidate boxes with the same predicted category excluding *b_i_*; IoU(*b_i_,b_j_*) ∈ [0,1] stands for the intersection over union of the two candidate boxes. If there are no other candidate boxes of the same category for candidate box i, then *d_i_*= 0. Since *d_i_*is calculated as the cumulative IoU value, its magnitude is affected by the number of same-category candidate boxes. Therefore, normalization is performed before computing the adaptive threshold. Let *n_i_*be the number of same-category candidate boxes excluding *b_i_*, then the normalized local density of the candidate box is expressed as:

(29) $${ℒ}_{i}=\frac{d_{i}}{max\left(n_{i},\;1\right)}$$

After obtaining the normalized local density factor, an IoU suppression threshold is calculated separately for each candidate box.

(30) $$T_{i}=\max\left(T_{\min},\min\left(T_{\max},T_{\text{base}}-\beta \cdot {ℒ}_{i}\right)\right)$$

Where *T_i_* ∈ [0,1] denotes the adaptive IoU threshold of candidate box *b_i_*; *T*_min_ and *T*_max_ represent the lower and upper bounds of the threshold respectively; *T*_base_ is the base threshold; *β* ≥ 0 refers to the density adjustment coefficient, which controls the influence strength of local density on the threshold. These thresholds satisfy 0 ≤ *T*_min_ ≤ *T*_base_ ≤ *T*_max_ ≤ 1. As the normalized local density factor ${ℒ}_{i}$ increases, the threshold *T_i_* of the candidate box decreases, enabling more candidate boxes to be suppressed. When the overlap of same-category boxes surrounding the candidate box is weak, *T_i_* approaches the base threshold and the suppression effect is weakened. In this work, *T*_base_ = 0.45, *T*_min_ = 0.30, *T*_max_ = 0.70, and *β* = 0.20.

2. Candidate Box Filtering in Stage I

After acquiring the suppression threshold *T_i_*for each candidate box, density-aware adaptive NMS is adopted to implement preliminary filtering on the candidate box set, and the filtered box set $\tilde{B}$ is obtained.

(31) $$\tilde{B}=N\left(B,\;T_{i}\right)$$

Where *B* denotes the original candidate box set entering the first stage of QHA-NMS; *N*(·) represents the category-aware NMS operation; $\tilde{B}$ is the candidate box set preserved after Stage I. In Stage I, all candidate boxes are first sorted in descendinge order according to the classification confidence *S*_conf_*_,i_*. Afterwards, pairwise comparisons are conducted between candidate boxes of the same category. Assume that two candidate boxes belonging to the same category possess thresholds *T_i_* and *T_j_* respectively. We take min(*T_i_, T_j_*) as the practical suppression threshold for this pair of candidate boxes. If two candidate boxes share the same category and IoU(*b_i_,b_j_*) *>* min(*T_i_, T_j_*), the candidate box with the lower sorting score will be eliminated. Candidate boxes with different categories do not suppress each other. The preserved set $\tilde{B}$ together with the computed thresholds *T_i_*are forwarded to subsequent processing from Stage I.

Stage II:

1. Multi-Dimensional Quality Perception

Stage I removes redundant candidate boxes mainly based on classification confidence and the overlap relationship between candidate boxes. Considering that classification confidence cannot fully characterize the spatial reliability of candidate boxes, this paper proposes the concept of candidate box quality vector to evaluate the quality of each candidate box.

(32) $$q_{i}=\left[S_{\text{conf},i},\;Q_{\text{IoU},j},\;Q_{\text{bound},i}\right]$$

Where *q_i_*denotes the quality vector of candidate box *b_i_*; *S*_conf_*_,i_*∈ [0,1] represents the classification confidence; *Q*_IoU_*_,j_* ∈ [0,1] stands for the localization-related prediction consistency, where subscript j refers to the candidate box in the original candidate set that shares the same category as *b_i_*and achieves the maximum IoU with *Q*_bound_*_,i_* ∈ [0,1] indicates the morphological quality of the candidate box.

Logarithmic normalization can convert metrics with disparate numerical scales into comparable dimensionless scalars (Zavadskas and Turskis, 2008). In addition, the logarithmic aspect ratio can characterize the multiplicative deviation of the actual aspect ratio relative to the reference aspect ratio (Michel et al., 2013). Given that candidate boxes of maize seedlings at the same growth stage follow certain aspect ratio patterns, inspired by the above methods, this work calculates the morphological quality *Q*_bound_*_,i_* of candidate boxes based on the deviation between the candidate box aspect ratio and the ideal aspect ratio. The calculation formula is given as follows:

(33) $$Q_{\text{bound},i}=\lambda \left[1-\frac{\left|\text{ln}\left(AR_{i}/AR_{\text{ideal}}\right)\right|}{\text{ln}\left(AR_{\text{max}}\right)}\right]$$

Where *AR_i_* > 0 denotes the aspect ratio of candidate box *b_i_*; *AR*_ideal_ *>* 0 is the predefined ideal aspect ratio; *AR*_max_ > 0 represents the maximum allowable aspect ratio deviation; *λ* ≥ 0 refers to the adjustment coefficient for morphological quality. In this work, *AR*_ideal_ = 1.5, *AR*_max_ = 5.0 and *λ* = 1.0. The morphological quality becomes higher when the aspect ratio of the candidate box approaches *AR*_ideal_.

2. Quality-Aware Candidate Box Reordering and Filtering

Tan et al. (2019) linearly fuse classification scores and IoU-based ranking scores to improve the ordering of candidate boxes. Chen et al. (2023) further verified the effectiveness of weighted fusion between classification confidence and IoU scores. Meanwhile, Boundary IoU demonstrates that boundary quality can provide supplementary evaluation information different from region overlap (Cheng et al., 2021). Inspired by the above studies, we integrate classification confidence, localization consistency and boundary quality, and perform weighted fusion on the three components of *q_i_*to obtain the comprehensive quality ranking score for the current candidate box.

(34) $$Q=w_{1}\times S_{\text{conf},i}+w_{2}\times Q_{\text{IoU},j}+w_{3}\times Q_{\text{bound},i}$$

Where *Q* ∈ [0,1] denotes the comprehensive quality score of the candidate box; *w*_1_, *w*_2_ and *w*_3_ represent the fusion weights corresponding to classification confidence, localization-related prediction consistency and morphological quality, respectively. All weights are non-negative constants and satisfy *w*_1_+*w*_2_+*w*_3_ = 1. In this work, the weights are set as *w*_1_ = 0.60, *w*_2_ = 0.30 and *w*_3_ = 0.10.

After obtaining the comprehensive quality score Q, Stage II reorders the candidate boxes retained from Stage I in descending order of Q, and adopts the IoU suppression thresholds *T_i_*derived in Stage I. The practical comparison threshold for each candidate box pair remains min(*T_i_,T_j_*). Finally, the qualityoptimized candidate box set B^*^ is obtained, where $B^{*}\subseteq \tilde{B}$. Since the candidate boxes preserved after Stage I already comply with the same-category constraint, the primary function of Stage II is to adjust the output priority. When the number of candidate boxes exceeds the maximum output limit, candidate boxes with higher comprehensive quality scores are preferentially retained. By default, Q is only utilized for sorting; the original classification confidence *S*_conf_*_,i_*is preserved in the final detection results, and Q will not replace the output confidence.

## 3 Results

### 3.1 Experimental configuration

All experiments were implemented using the PyTorch framework and Ultralytics YOLOv8 (v8.0.49). The input image size was set to 640×640, and all models were trained for 200 epochs with a batch size of 16 and an early-stopping patience of 50. AdamW was adopted as the optimizer, with a weight decay of 5 × 10^−4^ and a warm-up period of three epochs. The initial learning rates were set to 1×10^−2^ and 5×10^−3^ for the two training stages, respectively. Data augmentation included HSV transformation, translation, scaling, horizontal flipping, and Mosaic augmentation, with HSV-H, HSV-S, HSV-V, degrees, translate, scale, FlipLR, and Mosaic set to 0.015, 0.7, 0.4, 5.0, 0.1, 0.5, 0.5, and 1.0, respectively. The same post-processing settings were applied in both the detection and counting experiments to ensure consistency between the two evaluations. All comparative experiments are fairly tuned under identical data augmentation strategies, optimizer configurations, input resolutions, training epochs and early stopping criteria. To evaluate the stability of the model, we conduct experiments with seven different random seeds: 0, 1, 2, 42, 100, 200 and 500. For each seed, the model is trained independently under identical experimental configurations. Furthermore, all computational tasks were executed on a high-performance workstation equipped with an NVIDIA RTX 4090 GPU. The software environment comprised Python 3.8.20, PyTorch 1.13.1, and CUDA 11.7. Comprehensive software configurations are summarized in **Table 2**.

**Table 2:** Hyperparameter settings and training configuration.

Training configuration	DAQ-YOLO
Epochs	200
Optimizer	AdamW
Batch size	16
Weight decay	5×10^−4^
Warm-up epochs	3
Initial learning rate	1×10^−2^ (Stage 1)/5×10^−3^ (Stage 2)
Input image size	640×640
Patience	50
Data augmentation	HSV-H: 0.015, HSV-S: 0.7, HSV-V: 0.4, Degrees: 5.0, Translate: 0.1, Scale: 0.5, FlipLR: 0.5, Mosaic: 1.0

### 3.2 Evaluation metrics

To rigorously validate the performance of the proposed DAQ-YOLO model, comprehensive training and testing were conducted on the self-constructed maize seedling dataset. In this paper, the following key evaluation metrics are used to quantitatively analyze the model performance. Specifically, mean Average Precision (mAP), including mAP50 and mAP50-95, was employed to characterize the detection robustness across diverse Intersection over Union (IoU) thresholds. Where: mAP50 refers to the mean average precision across all categories when the IoU threshold is set to 0.5; mAP50–95 denotes the average of multi-threshold mAP values calculated with IoU thresholds ranging from 0.5 to 0.95 at an interval of 0.05. Precision (*P*) and Recall (*R*) were utilized to evaluate the detector’s discriminative power and ability to mitigate omissions, respectively. Giga Floating-Point Operations (GFLOPs), total Parameters, Latency, and frames per second (FPS) were calculated to assess the model’s efficiency. In addition to the above metrics, Mean Absolute Error (MAE), Root Mean Squared Error (RMSE), Mean Absolute Percentage Error (MAPE), Spearman correlation coefficient (*r_s_*) are also adopted to evaluate the counting error of the model and investigate the correlation between predicted counts and ground-truth values.

For ablation experiments and model comparison experiments, results are reported in the form of mean ± standard deviation, which are calculated based on seven repeated experiments with different random seeds for each run. The curves of P, R, mAP50 and mAP50-95, as well as representative visualized detection results, are generated using the model trained with random seed = 0.

### 3.3 Ablation study

To systematically evaluate the individual and synergistic contributions of the three proposed modules within the DAQ-YOLO architecture, a series of ablation experiments were performed. The results are summarized in **Table 3**.

**Table 3:** Results of the ablation study.

D-EMA	AQFL	QHA-NMS	P/%	R/%	mAP50/%	mAP50-95/%	GFLOPS	Parameters	Latency/ms
—	—	—	87.0 ± 0.9	82.8 ± 0.8	88.6 ± 0.7	48.0 ± 0.7	8.1	3.01M	15.9 ± 0.6
√			87.6 ± 0.6	83.2 ± 0.5	89.0 ± 0.5	49.4 ± 0.7	8.3	3.17M	16.0 ± 0.7
	√		88.9 ± 0.7	84.1 ± 0.5	89.4 ± 0.6	49.6 ± 0.9	8.1	3.01M	16.0 ± 0.7
		√	88.9 ± 0.8	85.1 ± 0.7	89.9 ± 0.6	55.1 ± 1.0	8.1	3.01M	16.2 ± 0.9
		(AdaptiveNMS)	86.7 ± 0.7	84.5 ± 0.6	89.0 ± 0.6	48.2 ± 0.8	8.1	3.01M	16.1 ± 0.7
		(QualityNMS)	88.7 ± 0.6	84.0 ± 0.5	88.8 ± 0.5	50.3 ± 0.8	8.1	3.01M	16.6 ± 0.8
√	√		88.9 ± 0.6	83.7 ± 0.5	89.2 ± 0.5	49.8 ± 0.7	8.3	3.17M	16.2 ± 0.7
**√**	**√**	**√**	**89.0 ± 0.9**	**86.1 ± 0.2**	**90.7 ± 0.6**	**56.0 ± 1.1**	**8.3**	**3.17M**	**17.3 ± 0.8**

Bold values indicate the results of the complete DAQ-YOLO model.

Analysis of the D-EMA Module: The D-EMA module leverages dynamic channel grouping and parallel spatial–contextual modeling to effectively mitigate the feature dilution of small-scale targets in dense environments. As indicated in **Table 3**, the individual integration of D-EMA yielded a substantial improvement in mAP50-95, rising from 48.0% to 49.4% (a 2.9% relative increase), while mAP50 increased from 88.6% to 89.0% (+0.5%), the parameter count increased by merely 0.16 M (from 3.01 M to 3.17 M), and the computational complexity, measured in GFLOPs, increased by only 2.5% (from 8.1 to 8.3 GFLOPs).

Analysis of the AQFL Module: When integrated individually, the AQFL module enhanced mAP50–95 from 48.0% to 49.6%(+3.3%) and mAP50 from 88.6% to 89.4% (+0.9%). By dynamically down-weighting easy-to-classify samples, this loss function compels the model to prioritize hard-to-classify candidates, thereby effectively alleviating the foreground-background class imbalance. The concomitant improvement in Precision (rising from 87.0% to 88.9%, +2.5%) underscores the module’s efficacy in suppressing background false positives and bolstering the reliability of detection confidence scores. Notably, these gains in discriminative power are achieved without incurring additional computational overhead during the inference phase.

Analysis of the QHA-NMS Module: To validate the efficacy of the proposed QHA-NMS, two single-stage variants were established as baselines: AdaptiveNMS (density-aware thresholding only) and QualityNMS (quality-aware scoring only). As summarized in **Table 3**, the individual application of AdaptiveNMS yielded a 0.4% improvement in mAP50–95 over the baseline, while QualityNMS achieved 4.8% over the baseline. In stark contrast, QHA-NMS—leveraging its heterogeneous cascaded architecture— achieved a coarse-to-fine collaborative optimization. This cascaded design propelled mAP50–95 to 55.1%, representing a substantial 9.5% leap over the best single-stage variant (QualityNMS) and a 14.8% surge relative to the baseline. Remarkably, this two-stage cascade incurred negligible computational overhead, demonstrating that the heterogeneous strategy provides superior accuracy gains at equivalent cost. These results further validate the inherent complementarity between density-aware thresholding and quality-aware scoring: the former mitigates spatial density heterogeneity, while the latter addresses the misalignment between classification and localization, thereby confirming that both components are indispensable for achieving optimal performance.

As can be seen from the processing latency metrics, the processing latency increases after adding the three modules sequentially. However, the maximum latency rises by only 1.4 ms (from 15.9 to 17.3).

**Figure 8:**
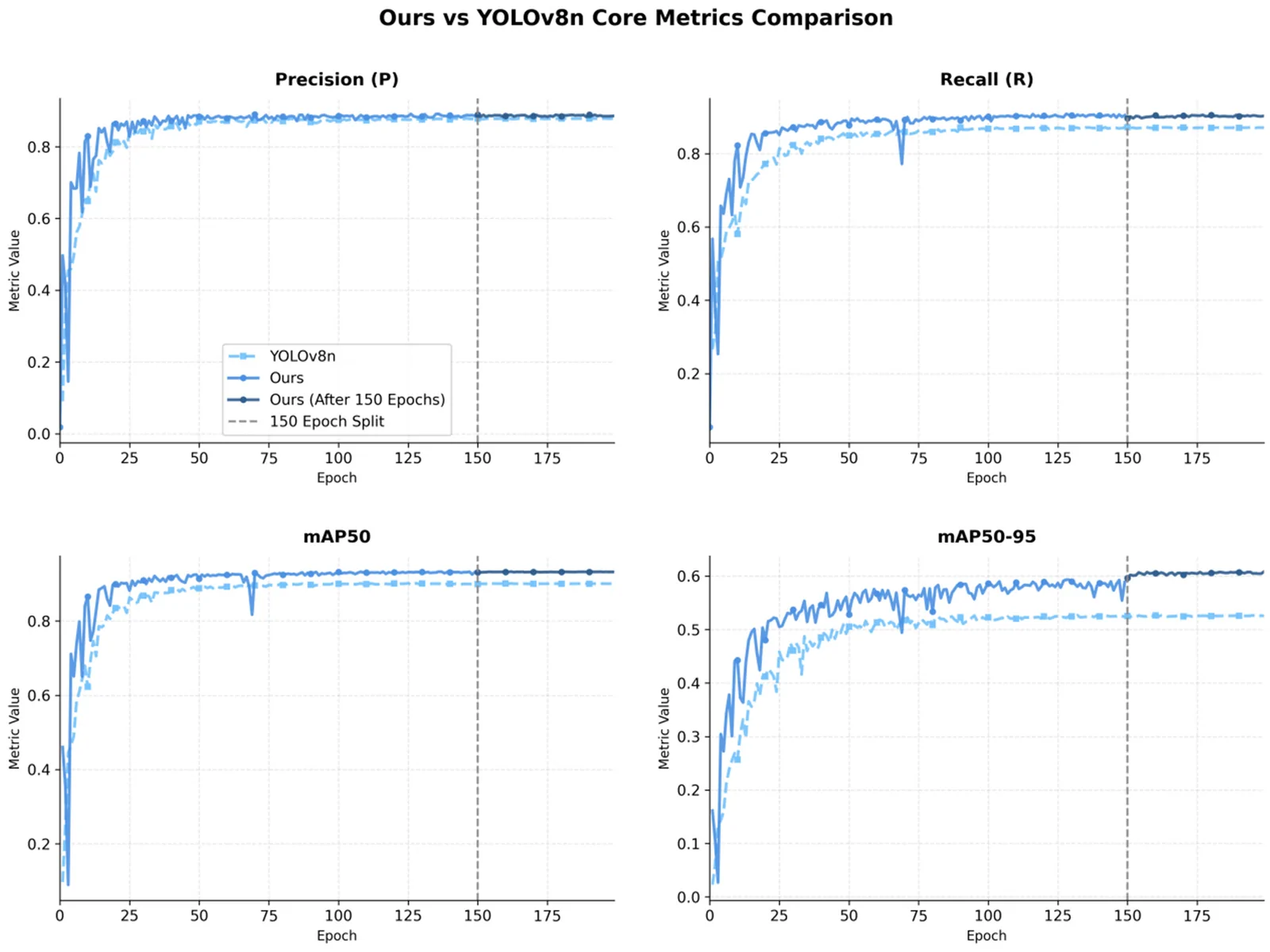
Comparison of performance metrics between DAQ-YOLO and YOLOv8n.

To verify the performance advantages of the proposed enhancements, a comparative training session was conducted over 200 epochs using YOLOv8n as the baseline model. The evolution of the core detection metrics is illustrated in **Figure 8**.

During the initial training phase (0–25 epochs), the proposed DAQ-YOLO demonstrated a significantly higher ascent rate in all performance metrics compared to the baseline, entering the convergence regime noticeably earlier and underscoring its superior feature-learning efficiency. In the intermediate stage (25–150 epochs), the proposed model consistently maintained a clear margin over YOLOv8n across Precision, Recall, mAP50, and mAP50-95. Beyond 150 epochs, while the baseline model entered a performance plateau, our improved framework continued to exhibit marginal gains without showing clear signs of overfitting. Ultimately, DAQ-YOLO achieved comprehensive superiority across the four core metrics. Most notably, the most substantial improvement was observed in mAP50-95, the definitive indicator of integrated detection robustness, thereby validating the dual optimization effect of our proposed strategies on both semantic classification accuracy and spatial localization precision.

### 3.4 Comparative experiments

#### 3.4.1 Comparative experiments of different NMS method

To evaluate the effectiveness of the proposed QHA-NMS method in this paper, we conduct NMS ablation experiments comparing this method with other mainstream post-processing approaches, including Soft-NMS, DIoU-NMS and WBF-NMS. In the ablation and comparative experiments on NMS variants, YOLOv8n was adopted as the baseline detection model for all schemes, with identical training weights and hyperparameters utilized consistently. The candidate boxes output by the model were fed into different NMS processing modules, respectively. Accordingly, the performance discrepancies among Soft-NMS, DIoU-NMS, WBF-NMS and QHA-NMS do not arise from retraining the detection model, but are mainly caused by differences in candidate box sorting and suppression strategies. The comparison results are shown in **Table 4**.

**Table 4:** Comparative experiments of different NMS method.

Models	P/%	R/%	mAP50/%	mAP50-95/%	GFLOPS	Parameters	FPS
YOLOv8n-Soft-NMS	87.2 ± 0.9	84.8 ± 0.7	87.8 ± 0.6	51.1 ± 0.9	8.1	3.01M	60.4 ± 2.2
YOLOv8n-DIoU-NMS	86.3 ± 0.7	82.1 ± 0.5	88.1 ± 0.5	51.7 ± 0.7	8.1	3.01M	61.7 ± 2.5
YOLOv8n-WBF-NMS	83.0 ± 0.9	82.2 ± 0.8	85.1 ± 0.8	47.7 ± 0.9	8.1	3.01M	55.7 ± 3.4
**YOLOv8n-QHA-NMS**	**88.9 ± 0.8**	**85.1 ± 0.7**	**89.9 ± 0.6**	**55.1 ± 1.0**	**8.1**	**3.01M**	**61.7 ± 3.1**

Bold values indicate the results obtained with QHA-NMS.

As can be observed from the experimental results, under the condition of identical model parameters and floating-point operations, the QHA-NMS method achieves substantially better performance than DIoUNMS and WBF-NMS. Compared with Soft-NMS, QHA-NMS improves mAP50 and mAP50–95 by 2.1% and 4.0%, respectively, while maintaining comparable Precision (P) and Recall (R). This demonstrates that, relative to other NMS variants, QHA-NMS can localize objects more accurately at high confidence thresholds, which further facilitates the overall performance improvement of object detection.

#### 3.4.2 Comparative experiments of different models

To comprehensively assess the competitive standing of our proposed framework, DAQ-YOLO was benchmarked against several one-stage detectors and various representative iterations of the YOLO series (Wang et al., 2024b; Jin et al., 2025; Chaman et al., 2026). The quantitative comparisons are detailed in **Table 5**.

**Table 5:** Comparison of performance with other models.

Models	P/%	R/%	mAP50/%	mAP50-95/%	GFLOPS	Parameters	FPS
YOLOv3t	83.4 ± 1.0	76.3 ± 0.4	81.4 ± 0.5	36.6 ± 0.7	13.0	8.67M	39.6 ± 1.5
YOLOv5n	86.5 ± 0.7	82.9 ± 0.5	87.3 ± 0.6	46.1 ± 1.4	4.2	1.77M	122.5 ± 7.6
YOLOv7t	90.3 ± 0.9	88.8 ± 0.7	92.1 ± 0.8	49.9 ± 1.0	13.2	6.01M	39.0 ± 1.5
YOLOv8n	87.0 ± 0.9	82.8 ± 0.8	88.6 ± 0.7	48.0 ± 0.7	8.1	3.01M	62.9 ± 2.7
YOLOv9t	85.1 ± 0.7	85.9 ± 0.7	89.5 ± 0.7	49.3 ± 0.9	7.7	2.62M	66.8 ± 3.5
YOLOv10n	88.3 ± 0.4	87.4 ± 0.4	92.0 ± 0.8	53.1 ± 1.0	6.5	2.27M	79.1 ± 3.8
YOLOv11n	88.8 ± 0.7	87.2 ± 0.6	92.0 ± 0.8	53.0 ± 0.8	6.3	2.58M	81.6 ± 4.9
YOLOv12n	88.1 ± 1.0	86.6 ± 0.3	91.4 ± 0.6	53.5 ± 1.3	5.8	2.51M	88.7 ± 3.2
SSD300 VGG16	90.8 ± 0.7	60.5 ± 0.4	64.0 ± 0.4	28.8 ± 0.7	62.7	41.72M	8.2 ± 0.5
Faster-R-CNN ResNet50	70.3 ± 0.6	69.5 ± 0.6	71.9 ± 0.6	39.7 ± 0.7	134.7	26.33M	3.6 ± 0.2
**Ours**	**89.0 ± 0.9**	**86.1 ± 0.2**	**90.7 ± 0.6**	**56.0 ± 1.1**	**8.3**	**3.17M**	**57.8 ± 2.8**

Bold values indicate the results of the proposed DAQ-YOLO model.

As shown in the table, although the proposed DAQ-YOLO model is slightly inferior to YOLOv10n, YOLOv11n and YOLOv12n in terms of Recall (R) and mAP50, it outperforms all the above models on Precision (P) and mAP50-95. Specifically, the mAP50–95 metric of DAQ-YOLO exceeds those models by 2.9%, 3.0% and 2.5%, respectively. This further verifies the superiority of the DAQ-YOLO model under high confidence thresholds.

Furthermore, when compared with the classic two-stage detector Faster R-CNN, the proposed DAQYOLO surpassed it by 41.0% in mAP50-95 (56.0% vs 39.7%). Remarkably, our method achieved a 16.2 fold increase in computational efficiency while slashing the parameter count by 87.9% (3.17 M vs 26.33 M). Relative to SSD300, DAQ-YOLO yielded a vibrant 94.4% improvement in mAP50–95 and an 86.8% reduction in computational overhead. These findings demonstrate that through targeted post-processing refinement, the proposed model can attain superior precision without compromising real-time performance. This underscores that the cost-effectiveness of algorithmic post-processing significantly outweighs the marginal gains typically associated with increasing architectural complexity.

To further evaluate the counting accuracy performance of the models, we calculate metrics including Mean Absolute Error (MAE), Mean Squared Error (MSE), Root Mean Squared Error (RMSE), and Mean Absolute Percentage Error (MAPE) (Chicco et al., 2021) for different models on the identical test set. Meanwhile, the Spearman’s rank correlation coefficient *r_s_*is adopted to analyze the correlation between predicted counts and ground-truth values. The results are presented in **Table 6**.

**Table 6:** Comparison of computing performance with other models.

Models	Total ground truth	Total predictions	MAE	MSE	RMSE	MAPE	*r_s_*
YOLOv3t	12338	13498 ± 305	12.81 ± 0.10	262.06 ± 1.28	16.19 ± 0.04	0.114 ± 0.006	0.816 ± 0.009
YOLOv5n	12338	13002 ± 203	7.69 ± 0.02	96.96 ± 1.03	9.85 ± 0.05	0.072 ± 0.003	0.876 ± 0.016
YOLOv7t	12338	12682 ± 153	4.86 ± 0.08	38.42 ± 0.31	6.20 ± 0.03	0.045 ± 0.001	0.924 ± 0.019
YOLOv8n	12338	11918 ± 167	4.84 ± 0.03	39.44 ± 0.38	6.28 ± 0.02	0.044 ± 0.003	0.942 ± 0.003
YOLOv9t	12338	12564 ± 145	4.29 ± 0.02	31.38 ± 0.34	5.60 ± 0.03	0.040 ± 0.001	0.944 ± 0.007
YOLOv10n	12338	12490 ± 128	3.89 ± 0.02	26.02 ± 0.20	5.10 ± 0.02	0.036 ± 0.001	0.918 ± 0.040
YOLOv11n	12338	12432 ± 137	3.75 ± 0.01	24.02 ± 0.24	4.90 ± 0.03	0.034 ± 0.001	0.943 ± 0.020
YOLOv12n	12338	12515 ± 141	4.10 ± 0.02	27.55 ± 0.14	5.25 ± 0.01	0.037 ± 0.002	0.937 ± 0.020
SSD300 VGG16	12338	10946 ± 352	15.89 ± 0.02	400.09 ± 3.87	20.00 ± 0.10	0.147 ± 0.004	0.712 ± 0.032
Faster-R-CNN ResNet50	12338	11518 ± 289	10.60 ± 0.27	204.67 ± 1.35	14.31 ± 0.05	0.099 ± 0.003	0.804 ± 0.008
**Ours**	**12338**	**12299 ± 105**	**2.90 ± 0.02**	**14.54 ± 0.29**	**3.81 ± 0.04**	**0.026 ± 0.002**	**0.954 ± 0.022**

Bold values indicate the results of the proposed DAQ-YOLO model.

As shown in **Table 6**, the test set contained 12,338 ground-truth maize seedlings, while DAQ-YOLO predicted 12,299 seedlings, resulting in a cumulative undercount of 39 seedlings. Among all the compared models, DAQ-YOLO produced the total prediction closest to the ground-truth count. In comparison, YOLOv8n yielded a cumulative counting error of 420 seedlings. In terms of image-level counting errors, DAQ-YOLO achieved the lowest MAE, MSE, RMSE, and MAPE, with values of 2.90, 14.54, 3.81, and 0.026, respectively. These results indicate that the model produced an average absolute counting error of approximately three seedlings per image, demonstrating its greater stability in controlling counting deviations across most test images. Compared with the YOLOv8n baseline, the MAE decreased from 4.84 to 2.90, while the MAPE decreased from 0.044 to 0.026, corresponding to reductions of approximately 40.1% and 40.9%, respectively. These improvements further confirm the effectiveness of the proposed strategies in enhancing maize seedling counting accuracy. In addition, SSD300-VGG16 and Faster RCNN produced MAE values of 15.89 and 10.60 and RMSE values of 20.00 and 14.31, respectively, which were substantially higher than those of the more recent YOLO-based models and DAQ-YOLO. These results suggest that conventional two-stage detectors are more susceptible to missed detections in dense scenes involving occlusion and small-scale seedlings, thereby leading to larger counting errors. Overall, DAQ-YOLO can estimate the number of maize seedlings more reliably in complex and dense scenarios, providing technical support for efficient seedling counting.

#### 3.4.3 Performance comparison of the model under different densities

To validate the model performance under varying density conditions, test images were categorized into three groups (low density, medium density, and high density) based on the aggregation of maize seedlings within each image instead of seedling counts. We calculate P, R, mAP50, mAP50-95, MAE, RMSE and Spearman’s rank correlation coefficient r_s_ for each group. The statistical results are listed in **Table 7**.

**Table 7:** Performance comparison of the model under different densities.

Models	Degree of density	Total images	Total ground truth	P/%	R/%	mAP50/%	mAP50-95/%	MAE	RMSE	*r_s_*
YOLOv8n	Low	37	3957	87.6 ± 0.6	85.0 ± 0.5	90.1 ± 0.6	51.1 ± 0.5	3.51 ± 0.03	4.45 ± 0.02	0.957 ± 0.018
Ours	Low	37	3957	90.2 ± 0.7	90.0 ± 0.2	92.2 ± 0.4	58.2 ± 0.7	1.89 ± 0.01	2.41 ± 0.03	0.981 ± 0.011
YOLOv8n	Medium	36	3898	86.0 ± 0.9	83.0 ± 0.8	88.4 ± 0.7	48.5 ± 0.6	4.75 ± 0.01	6.00 ± 0.02	0.933 ± 0.025
Ours	Medium	36	3898	88.3 ± 0.8	88.0 ± 0.1	91.2 ± 0.7	56.1 ± 1.0	2.78 ± 0.02	3.50 ± 0.04	0.960 ± 0.023
YOLOv8n	High	37	4483	82.5 ± 1.1	79.5 ± 0.6	86.2 ± 0.7	45.6 ± 0.8	6.24 ± 0.03	7.90 ± 0.04	0.876 ± 0.017
Ours	High	37	4483	85.4 ± 1.0	83.5 ± 0.2	89.2 ± 0.5	54.1 ± 1.2	4.03 ± 0.02	5.05 ± 0.04	0.900 ± 0.018

As shown in **Table 7**, the detection and counting performance of both models gradually declined as maize seedling density increased, confirming that severe overlap and occlusion pose greater challenges to accurate seedling identification. Nevertheless, DAQ-YOLO consistently outperformed the YOLOv8n baseline across all density levels. In low density, medium density, and high density scenes, DAQYOLO improved mAP50–95 by 7.1, 7.6, and 8.5 percentage points, respectively, while reducing MAE by approximately 46.2%, 41.5%, and 35.4%. Notably, under high-density conditions, the proposed model increased Recall from 79.5% to 83.5% and the Spearman correlation coefficient from 0.876 to 0.9 indicating fewer missed detections and stronger agreement between predicted and ground-truth counts. These results demonstrate that the proposed D-EMA, AQFL, and QHA-NMS modules effectively alleviate feature dilution, localization errors, and redundant-box suppression in densely distributed seedling scenes, thereby improving the robustness and reliability of maize seedling counting.

#### 3.4.4 Visualization of detection results

**Figure 9:**
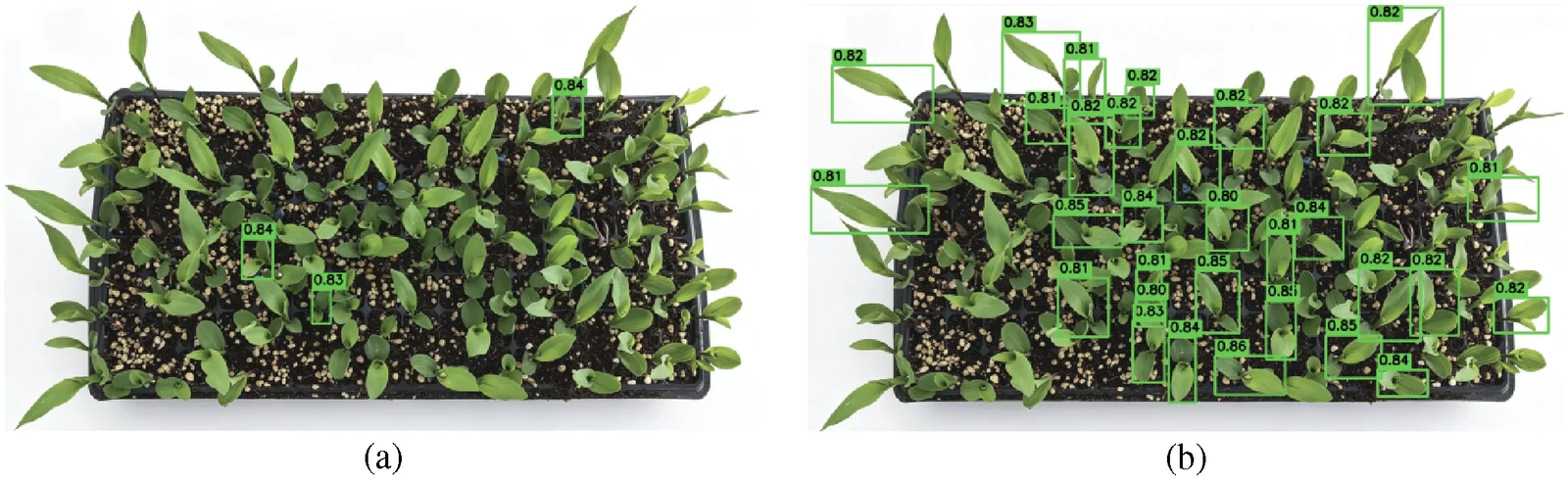
Maize seedling detection results: **(A)** YOLOv8n with threshold 0.8; **(B)** DAQ-YOLO with threshold 0.8.

**Figure 9** compares the detection performance of the YOLOv8n model and the proposed DAQ-YOLO.

The detection results reveal that both YOLOv8n and DAQ-YOLO suffer from missed detections under a high confidence threshold. Nevertheless, YOLOv8n produces significantly more missed detections than DAQ-YOLO, which further demonstrates the performance superiority of the proposed model under dense object scenarios and high confidence thresholds.

## 4 Discussion

Building upon the YOLOv8n baseline, this study proposes the DAQ-YOLO model. Addressing core challenges in maize seedling counting tasks such as dense small targets, severe occlusion, and low detection accuracy. Through the synergistic optimization of three core modules, D-EMA, AQFL and QHA-NMS, the detection performance is effectively improved while the number of parameters remains roughly unchanged. Regarding the efficacy of model optimization, the three proposed modules constitute an end-to-end optimization pipeline encompassing feature extraction, loss function formulation, and post-processing screening, wherein each module functionally complements the others. Specifically, the D-EMA module resolves the feature dilution predicament of small targets in dense scenarios through dynamic channel grouping combined with the parallel modeling of spatial and local contexts. Impressively, it yields a 2.9% increment in mAP50–95 while introducing a mere 0.16M additional parameters, thereby substantiating the efficacy of dynamic attention mechanisms in agricultural small target detection. Furthermore, the AQFL module optimizes the loss function from the perspective of coupling classification with localization. By incorporating a dynamic focusing parameter and an IoU-based quality weight, it effectively overcomes the issues of gradient inundation for challenging samples and the inherent decoupling between classification and localization. Consequently, this module markedly elevates detection precision and mitigates background false positives. As the core innovation in the post-processing phase, the QHA-NMS module transcends the limitations of fixed thresholds in traditional NMS via a two-stage strategy: densityaware adaptive threshold preliminary screening followed by multidimensional quality-aware fine-grained ranking. Without incurring any supplementary computational overhead, it propels the mAP50–95 by 14.8% relative to the baseline. Its performance substantially eclipses that of standalone Adaptive NMS or Quality NMS, demonstrating that the synergistic optimization of spatial density adaptation and multidimensional quality evaluation constitutes a pivotal strategy for rectifying missed detections and false positives among dense small targets.

From the perspective of practical application value, the proposed DAQ-YOLO model achieves an mAP5095 of 56.0% with only 3.17M parameters and a computational cost of 8.3 GFLOPs. Its performance outperforms not only classic two-stage detectors SSD300 and Faster R-CNN, but also advanced lightweight models including YOLOv9t, YOLOv10n, YOLOv11n and YOLOv12n. Such a lightweight design can effectively improve the counting efficiency of maize seedlings. It provides technical support for dynamic monitoring and accurate counting of maize seedlings in dense scenarios.

Although this study has yielded promising results, several limitations remain to be addressed. First, the experimental dataset comprises maize seedling images acquired within greenhouse environments, representing relatively uniform conditions. There is a conspicuous lack of samples from extreme open-field scenarios characterized by wind and rain, sudden illumination fluctuations, weed occlusion, and complex soil backgrounds. Consequently, the robustness of the model in natural field environments necessitates further empirical validation. Second, the current model solely accomplishes the detection and counting of maize seedlings; it has yet to integrate morphological traits, such as plant height, stem diameter and leaf count, for a comprehensive assessment of seedling conditions. Therefore, a gap persists between the current capabilities of the model and the full-chain requirement of smart agriculture, spanning from precise monitoring to intelligent decision-making.

## 5 Conclusions

To improve detection accuracy and reduce missed or false detections in dense seedling environments, this study proposes DAQ-YOLO—a lightweight maize seedling detection and counting model based on the enhanced YOLOv8n framework. By incorporating a Dynamic Efficient Multi-scale Attention (D-EMA) module, an Adaptive Quality-aware Focal Loss (AQFL), and a Quality-enhanced Heterogeneous Adaptive NMS (QHA-NMS) strategy, improvements were integrated into feature extraction, loss supervision, and post-processing stages. Experimental results on a self-collected maize seedling dataset demonstrate that the proposed model achieves favorable performance.

Although the proposed model achieves favorable performance, there remains further work to be carried out. Future research will be conducted from the following aspects:

1. Attempt to deploy the DAQ-YOLO model on UAVs, embedded vision terminals or inspection robots to realize real-time monitoring of maize seedling emergence status.
2. Extend the application scenarios of the DAQ-YOLO model to seedling detection of other densely planted crops (such as wheat, rice and rapeseed) and verify the generalization of the detection model.
3. Diversify the training data by collecting images under varying illumination, backgrounds and dense conditions in open-field environments, and enhance the model robustness via data augmentation and transfer learning.

## Data Availability

The raw data supporting the conclusions of this article will be made available by the authors, without undue reservation.
